# Healing of Chronic Wounds by Copper Oxide-Impregnated Wound Dressings—Case Series

**DOI:** 10.3390/medicina57030296

**Published:** 2021-03-22

**Authors:** Eyal Melamed, Patrick Kiambi, Dancan Okoth, Irena Honigber, Eran Tamir, Gadi Borkow

**Affiliations:** 1 Department of Orthopedics, Rambam Medical Center, Haifa 31096, Israel; 2 Dermatology Unit, Kenyatta National Hospital, Nairobi 00202, Kenya; kiambipatrick@gmail.com (P.K.); duncaneto@yahoo.com (D.O.); 3 Foot and Ankle Unit, Yitzhak Shamir Medical Center, Tzrifin 7073001, Israel; irenahon@gmail.com (I.H.); drtamir.foot@gmail.com (E.T.); 4 MedCu Technologies Ltd., Herzliya 4672837, Israel; gadib@medcu.com

**Keywords:** case series, copper oxide, dressings, granulation tissue, wound healing

## Abstract

Novel antimicrobial wound dressings impregnated with copper oxide micro-particles have been cleared for treatment of acute and chronic wounds. Our objective is to provide preliminary data regarding the potential benefit of using these novel wound dressings including in non-infected wounds. Methods involved the treatment of wounds that responded partially or poorly to conventional wound healing treatments with copper oxide impregnated wound dressings in patients with a range of etiologies. Ten cases of patients with etiologies such as diabetes mellitus, sickle cell disease, renal failure, and necrotizing fasciitis, in which the application of copper oxide impregnated wound dressings in infected and non-infected wounds, which resulted in significant enhanced wound healing, are presented. This was exemplified by clearing of the wound infections, reduction of the fibrous and/or necrotic tissue and by intense granulation, epithelialization, and wound closure. The described 10 case reports support our hypothesis that the copper oxide-containing wound dressing not only confers protection to the wound and the dressing from microbial contamination, and in some cases may help clear the wound infections, but in addition and more importantly, stimulate skin regeneration and wound healing. Our findings are in line with previous animal and in vitro studies showing that copper plays a key role in angiogenesis and skin regeneration. These case reports support the notion that the use of copper oxide impregnated wound dressings may be an important intervention in the arsenal of wound treatment modalities, especially in hard to heal wounds.

## 1. Introduction

Copper is a trace mineral essential for many wound healing-related processes [1,2]. Copper stimulates (a) angiogenesis [3] by upregulating Hypoxia Induced Factor 1alpha (HIF-1α) [4], vascular endothelial growth factor (VEGF) [5], and Cu-dependent transcription factor Atox1 [6]; (b) expression of integrin [7]; (c) stimulation of secretion of fibrinogen, elastin, and collagen by dermal fibroblasts [8,9] and their stabilization [10,11]; (d) upregulation of copper-dependent enzymes and polysaccharides, such as lysyl oxidase, metalloproteinases, glycosaminoglycans, and small proteoglycans, important for matrix remodeling, cell proliferation, and re-epithelization [12,13,14,15], and (e) migration of skin and stem cells [16,17].

Copper has also potent wide spectrum biocidal properties [18,19]. Copper ions, either alone or in copper complexes, have been used for centuries to disinfect liquids, solids, and human tissue [18]. The mechanisms of copper’s biocidal activity include alteration of microbial proteins and inhibition of their biological assembly and activity; plasma membrane permeabilization; and membrane lipid peroxidation [18]. In contrast to the antibiotic-resistant microbes that have evolved in less than 50 years of antibiotic use, copper-tolerant microbes are extremely rare due to the non-specific and parallel damage caused by copper to many key components of microorganisms [18].

The risk of adverse skin reactions due to copper exposure is extremely low [20,21]. Copper is not only considered safe to humans, as demonstrated by the widespread and prolonged use of copper intrauterine devices and copper food supplementation [22,23], but is an essential metal needed for normal metabolic processes [23]. Copper oxide impregnated medical devices and consumer products have been found to be safe in many studies [24,25,26]. 

Foot ulcers are among the most often occurring chronic wounds [27], with global prevalence of 6.3% of diabetic patients, affecting nearly 20 million people annually [28], leading to impaired mobility and amputation [29]. Aggressive wound care, consisting of infection control, sharp debridement, off weight bearing, and other basic approaches, often results in wound closure [30]. However, many chronic wounds fail to heal and novel treatments are needed. 

We have hypothesized that the incapacity of wounds to heal in individuals with diabetic ulcers, decubitus ulcers, peripheral vascular disease, or other wounds with compromised healing capacity, may be partially due to low copper levels in the wound site resulting from decreased blood supply [1]. We also hypothesized [1] that slow release of copper ions from wound dressings would not only reduce the risk of wound and dressing contamination, similarly to silver dressings, but more importantly, would also enhance wound repair especially in cases of diabetic ulcers, where the healing process is impaired. Angiogenesis and skin regeneration would be induced by the copper ions released from the wound dressings directly into the wound vicinity. 

We demonstrated the capacity of the copper oxide impregnated wound dressings to enhance wound closure in a diabetic (db/db) animal model [4]. Similar results of enhanced healing by copper compounds in wound healing animal and cell culture models were also reported and the incorporation of copper particles in wound dressing applications is increasingly explored [31,32,33,34,35].

Here we report the first clinical case series that demonstrates that continuous dermal application of the copper oxide-impregnated wound dressings on hard to heal, infected, and non-infected wounds, resulting in granulation formation and rapid wound closure. Some of the treated wounds failed or responded poorly to conventional treatments, indicating the significant potential of copper oxide-containing wound dressings (COD) to enhance wound healing.

## 2. Materials and Methods

### Copper Oxide Microparticles Impregnated Wound Dressings

**Figure 1:**
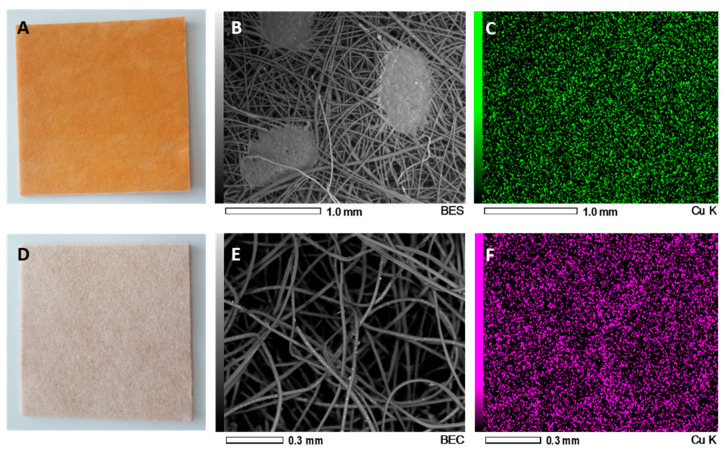
Scanning electron microscopy (SEM) of the copper oxide-containing wound dressing. The copper oxide wound dressing consists of two layers. A non-stick spunbond polypropylene layer (**A**) and a highly absorbent needle punch fabric (**D**). The non-stick polypropylene layer is put directly on the wound. Scanning electronic microscope (SEM) images (**B**,**E**) and energy-dispersive X-ray spectroscopy (EDS) analysis of each layer (**C**,**F**) demonstrate the presence of copper oxide microparticles impregnated in the fibers.

**Figure 2:**
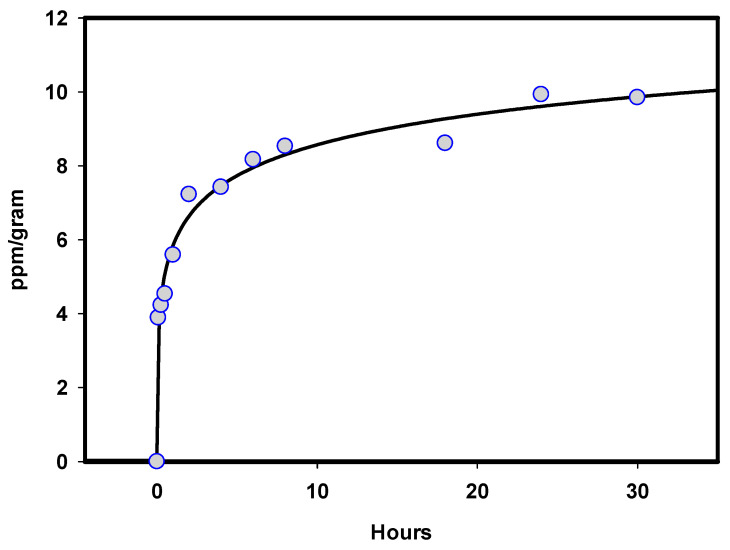
Copper ions elution from Copper oxide-containing wound dressings (COD). One-gram swatches of COD were incubated with saline at 37 °C between 5 min to 30 h. The amount of copper ions eluting to the saline solution was determined by colorimetry assay using bicinchoninic acid [38].

Recently, by using a platform technology that introduces copper oxide into fibers [36,37], wound dressings impregnated with copper oxide microparticles (COD, Figure 1) are produced. The use of these wound dressings for the treatment of acute and chronic wounds has been cleared by the FDA, EU, and the Israeli Ministry of Health. The dressings are sterile, soft, single use wound dressings composed of an absorbent layer for exudating wounds and one or two external spunbond nonwoven layer(s). The spunbond layer is placed directly on the wound. Both the absorbent and spunbond layers contain copper-oxide microparticles. The copper oxide microparticles serve as a reservoir of copper ions. The copper ions, which are constantly released from the copper oxide microparticles in ppm levels in the presence of wound moisture (Figure 2), endow the wound dressings with potent biocidal properties [26]. 

## 3. Results

### 3.1. Case Report 1 

**Figure 3:**
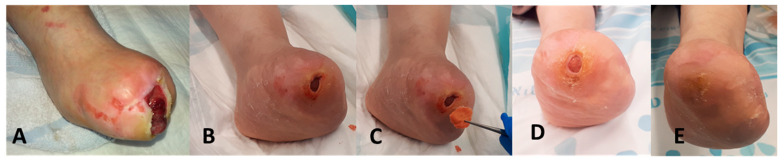
Closure of a 6-year indolent chronic wound in from insulin-dependent diabetes mellitus (IDDM) patient (Case Report 1). (**A**) The patient underwent a trans-metatarsal amputation in her left foot in 2013. (**B**) The wound was not closed for six years and in June 2019 the wound was about 7 mm deep with furrow (tunneling surrounding it). (**C**) On 27 June 2019 the wound was filled with COD wound dressing (WD). WD was changed every three days. (**D**) One week later the wound volume was reduced by approximately 90%. (**E**) On 10 August 2019, the wound was completely closed.

A 34-year-old female patient, suffering from insulin-dependent diabetes mellitus (IDDM) and neuropathy, had a trans-metatarsal amputation in her left foot (Figure 3A) on 19 December 2013. For six years the wound did not close despite standard of treatment (SOC), with occasional infectious episodes. On 24 June 2019 she was seen in the emergency room due to another infectious episode. She was prescribed antibiotics and seen in the clinic three days later. At that day the infection had resolved. The wound measured about 7 mm deep with furrow (tunneling surrounding it) (Figure 3B). The wound was packed with double layer COD (Figure 3C) for seven days. After a week the wound was filled with new tissue (~90% reduction in wound volume, Figure 3D). Weekly dressing changes were done by the patient at home or in the clinic by the attending nurse. At the follow up visit, six weeks after the initiation of the application of the COD dressings, the wound was completely closed (Figure 3E).

### 3.2. Case Report 2

**Figure 4:**
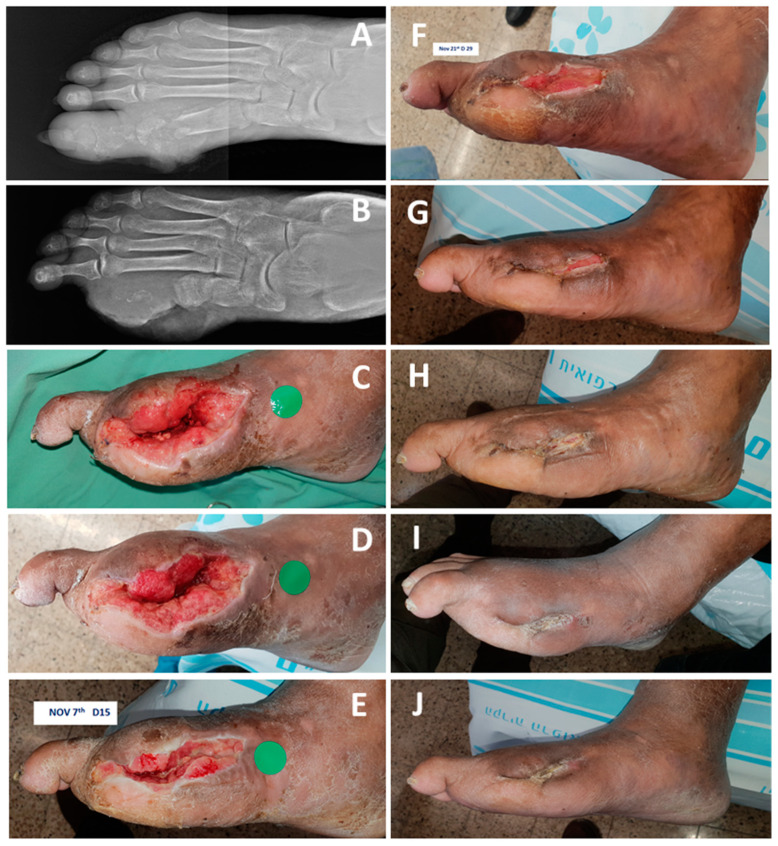
Closure of a wound in a non-insulin-dependent diabetes mellitus (NIDDM) patient (Case Report 2). (**A**)The patient suffered from osteomyelitis of the first ray in the right leg. (**B**) The patient underwent an amputation of the first ray on 9 October 2019. (**C**) After five days of standard of care (SOC) and two vacuum-assisted closure (VAC) sessions, COD was applied. (**D**) Four days after COD treatment the wound seemed to respond favorably to COD and same treatment was continued. (**E**) Fifteen days of COD treatment. (**F**) Twenty-nine days of COD treatment. (**G**) Forty-six days of COD treatment. (**H**) Fifty-seven days of COD treatment. (**I**) After 74 days of COD treatment the wound was completely closed, and the COD treatment was stopped. (**J**) The wound remained solidly closed as seen 94 days after the commencement of the COD treatment.

A 60-year-old male patient with non-insulin-dependent diabetes mellitus (NIDDM) with neuropathy, suffered from osteomyelitis of the right foot big toe and first metatarsal head (Figure 4A). He underwent amputation of the first ray (Figure 4B), after which the wound was treated with chlorine-based dressing and then two three-day sessions of two consecutive vacuum-assisted closure (VAC) treatments. Due to delay in approval of home VAC therapy, a temporary COD dressing was applied and in lieu of the good response it was continued (Figure 4D–H) until complete wound closure on day 74 (Figure 4I,J). 

### 3.3. Case Report 3

**Figure 5:**
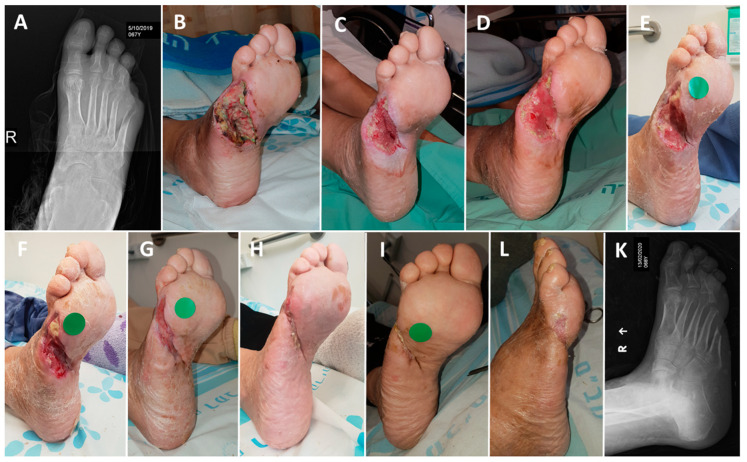
Closure of a wound in a NIDDM patient (Case Report 3). (**A**,**B**) The patient underwent right-leg fifth toe amputation due to osteomyelitis and continued infection on 17 October 2019. (**C**) After 20 days of SOC including 10 VAC sessions, the patient was discharged home on 7 November and the wound was covered with the COD. (**D**) Two days of COD treatment. (**E**) Ten days of COD treatment. (**F**) Seventeen days of COD treatment. (**G**) Thirty-one days of COD treatment. (**H**) Fifty-five days of COD treatment. (**I**) After 77 days of COD treatment the wound was completely closed and the COD treatment was stopped. (**L**,**K**) The wound remained solidly closed 20 days after wound closure.

A 68-year-old female suffering from type II diabetes (IDDM), neuropathy, and chronic obstructive pulmonary disease (COPD) was presented with osteomyelitis of the fifth metatarsal head with gangrene of the toe and infection along the flextor tendons. She underwent fifth ray amputation and debridement (Figure 5A,B). Bone cultures yielded pseudomonas and she was treated accordingly with meropenem for four weeks. The wound was treated initially with chlorine-based dressings for 2.5 weeks followed by 10 days of VAC therapy, which she refused to continue further due to inconvenience. Therefore, COD dressing was applied on the 28th post-operative day (Figure 5C). The antibiotic was stopped at that time. After 77 days of COD treatment the wound was completely closed (Figure 5I). The wound remained solidly closed as seen 20 days after wound closure (Figure 5L,K). 

### 3.4. Case Report 4

**Figure 6:**
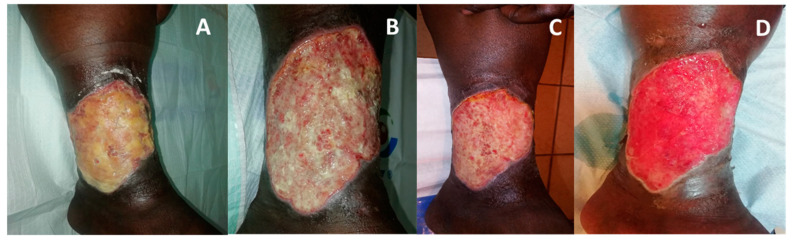
Dramatic improvement of a heavily infected venous ulcer (Case Report 4). (**A**) Upon patient arrival to the clinic, the heavily multidrug resistant *Escherichia coli* infected wound in supra-malleolar region underwent conservative desloughing and wound bed preparation. This was followed by COD application. (**B**) Two days after COD application. (**C**) Five days of COD application. (**D**) Seven days of COD application, resulting in intense granulation and edema reduction.

A 35-year-old female patient with sickle cell disease arrived at the clinic with a non-healing wound on the anterior aspect of distal right leg, which had been treated previously with many wound dressings, including silver sulfadiazine. Wound cultures revealed biofilm layer with multidrug resistant *Escherichia coli* colonizing the wound (expressed as a thick layer of fibrin in Figure 6A). Following conservative de-sloughing and wound bed preparation, the wound was covered with COD that was changed every 2–3 days. After seven days of COD treatment, the bacterial culture was negative, and there was a drastic reduction in edema and intense granulation tissue formation. 

### 3.5. Case Report 5

**Figure 7:**
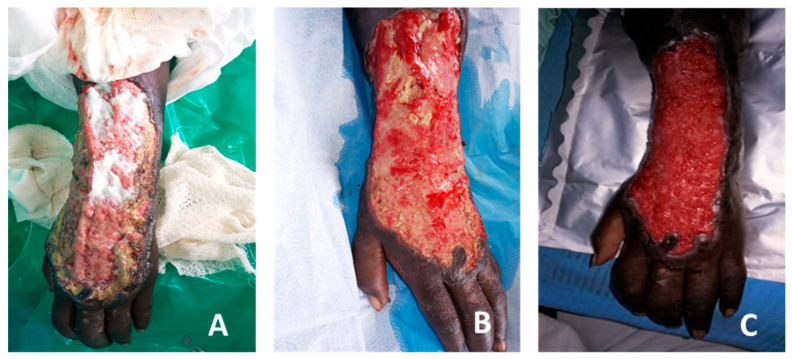
Dramatic improvement of a necrotizing fasciitis arm (Case Report 5). (**A**) Extensive loss of dorsal skin and subcutaneous tissue after necrotizing fasciitis from infected dialysis shunt. After one week of treatment with silver-based cream the wound has pale granulation tissue with lot of fibrin and necrotic tissue. (**B**) After two weeks of COD treatment large islands of red granulation tissue replace the necrotic and fibrinous tissue. (**C**) additional one week of COD results in full dense red granulation tissue. The wound is ready for skin grafting.

A 23-year-old male patient with renal failure arrived at the clinic with loss of skin and subcutaneous tissue due to necrotizing fasciitis of the forearm and wrist after his dialysis shunt had become infected (Figure 7). He had extensive necrotic and fibrinous tissue, which was treated initially with silver dressings for a week, without significant improvement. Hence, treatment was changed to COD, which were changed twice weekly. After three weeks of COD treatment, the wound was full with dense red granulation tissue, ready for skin grafting (Figure 7C).

### 3.6. Case Report 6

**Figure 8:**
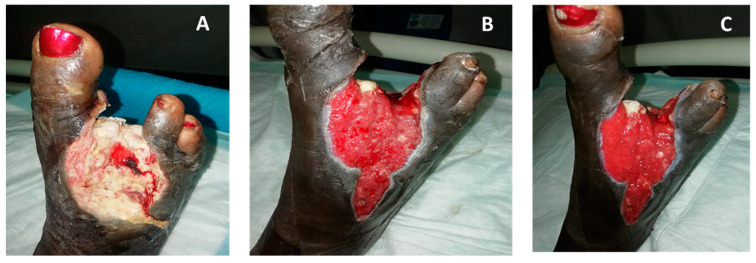
Dense granulation following surgery in a diabetic patient with peripheral vascular disease (PVD) (Case Report 6). (**A**) One day post-surgery, the wound started to be treated with COD. (**B**) Twelve days post-surgery, very significant granulation tissue was observed despite the PVD. (**C**) After 18 days of COD treatment the wound was ready for second tarsal nibbling and skin graft on dorsal foot.

A 45-year-old female with diabetes and peripheral vascular disease (PVD) underwent second and third toe ray amputation. Following surgery, the wound had profuse fibrous tissue (Figure 8A). COD was applied with dressing change every three days. Abundant granulation tissue was observed after 12 days (Figure 8B), and at 18 days the wound was ready for skin grafting (Figure 8C).

### 3.7. Case Report 7

**Figure 9:**
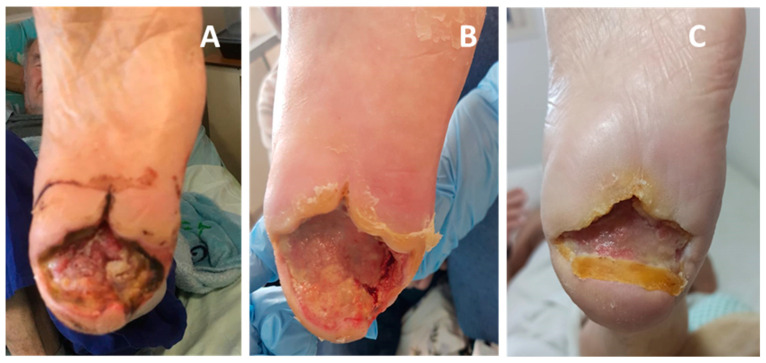
Exposed bone covered by granulation tissue in ischemic diabetic patient (Case Report 7). (**A**) Following angioplasty and surgical debridement, COD dressing was initiated. The dressing was replaced daily with a new COD. (**B**) Eighteen days following COD treatment, the exposed bone was covered by new tissue. (**C**) Three months following COD treatment, the wound was significantly smaller.

A 69-year-old patient with IDDM, end stage renal failure, and hemodialysis and severe PVD had critical left foot ischemia and wet gangrene of plantar and medial aspect of the heel. After a successful angioplasty, a surgical debridement was performed, all the necrotic tissue was removed, and the calcaneal bone was exposed. Antibiotic treatment with Vancomycin and Ertapenem was given for 10 days based on results of tissue culture. COD dressing was initiated four days following the surgical debridement (Figure 9A) and changed every day. After 18 days of COD dressing treatment, the wound bed improved, and the exposed bone was covered with granulation tissue (Figure 9B). Wound closure continued with significant reduction in wound size three months following COD treatment (Figure 9C). 

### 3.8. Case Report 8 

**Figure 10:**
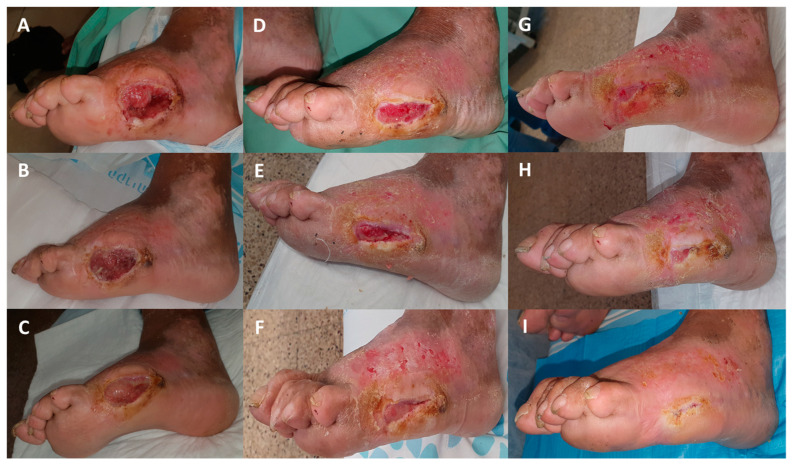
Enhanced wound healing after switching to COD treatment (Case Report 8). Sixty-year-old man with NIDDM underwent resection of the fifth metatarsal of the left foot due to chronic ulcer and osteomyelitis. Due to insufficient wound healing the patient underwent successful percutaneous angioplasty of the left leg. (**A**) One week after angioplasty. The patient was treated with standard dressings and VAC without significant progress. (**B**) Seven days later (14 days post angiography), no progress is observed. The wound measured 47 mm × 23 mm. Treatment with absorbent dressing was applied. (**C**) Six days later the wound size remained steady, measuring 46 mm × 23 mm (2.5% area reduction/week). (**D**) Nine days later, this time treatment was with COD. Wound size has been reduced to 43 mm × 13.5 mm (35% area reduction/week) with deep red granulation tissue. (**E–I**) Serial photos over additional two months of COD until final wound closure.

60-year-old man with NIDDM was admitted with osteomyelitis of the fifth ray of the left foot secondary to two years of nonhealing ulcer. Fifth metatarsal resection was carried out leaving the distal third of the metatarsal and the fifth toe. Cement spacer with antibiotics was used to stabilize the soft tissue and leash antibiotic locally. Due to delayed healing, the patient had undergone successful angioplasty two months after his admission. In the three weeks following angioplasty the wound was treated with various standard of care absorbent dressing without improvement (~2.5% wound area reduction per week) (Figure 10A). Copper oxide dressing was applied and documented 45% area reduction in the first nine-day interval (35% per week) (Figure 10D). Two months later the wound was closed (Figure 10I).

### 3.9. Case Report 9

**Figure 11:**
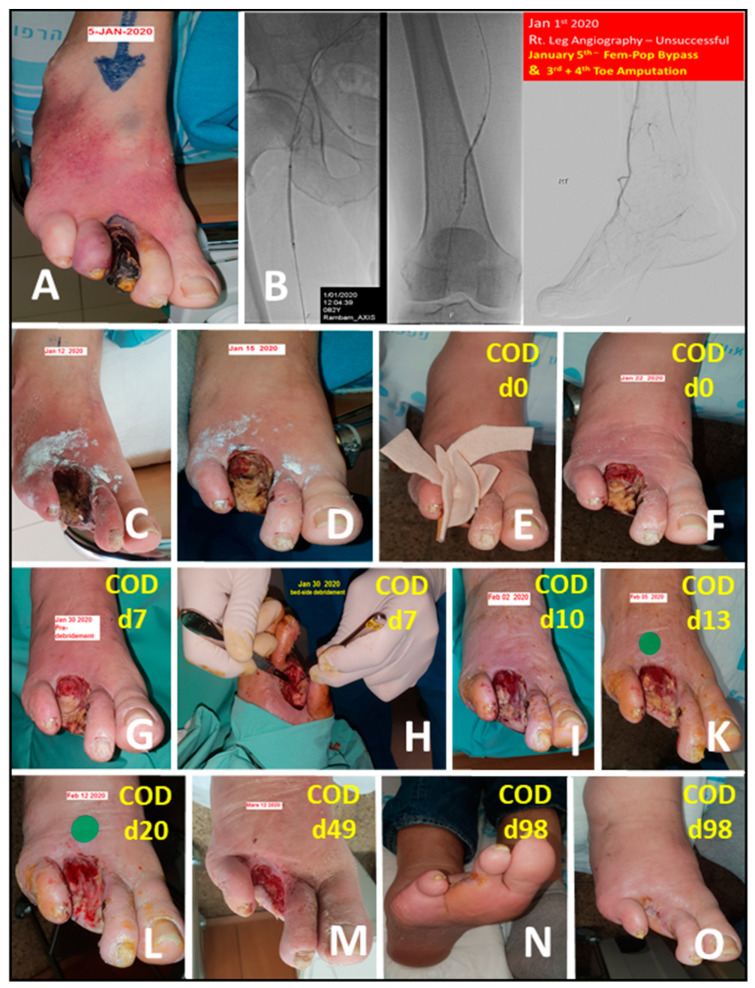
Wound closure following amputation due to gangrene in ischemic diabetic patient (Case Report 9). Eighty-two-year-old man with NIDDM and end stage renal disease (ESRD) on dialysis was admitted due to gangrene of the third toe and ischemia of the fourth one with cellulitis of the right foot (**A**). The patient had peripheral vascular disease (PVD) with intermittent claudication and no palpable pulses distal to the femoral. Attempted angiography was unsuccessful due to hard calcifications of the arteries (**B**). Subsequent femoral-popliteal bypass surgery was successful with good improvement of the blood flow and temperature to the level of the midfoot. The surgery was followed by third and fourth ray amputation in the same surgery. Following surgery the amputation wound was treated with antibiotic and twice a day chlorine based solution and was still ischemic (**C**,**D**). On the ninth day post-surgery antibiotic treatment was stopped and COD was begun (**D**,**E**, marked as COD d-0). One and two weeks later a progression or the granulation tissue is observed (**F**,**G**). For the most part the wound was still filled with necrotic tissue and bed side debridement was attempted with minor improvement (**H**). COD was used again and three days, one week, two weeks, and six weeks later further progression of the wound was seen (**I**,**K**–**M**). On a follow up visit, 14 weeks from the beginning of COD dressing, the wound was closed (**N**,**O**).

An 82-year-old patient with NIDDM and end stage renal disease (EDRD), on dialysis came with gangrene of the third toe, ischemia of the adjacent toe, and cellulitis on the right foot (Figure 11A). The patient had intermittent claudication and no pulses could be felt. Antibiotic treatment was begun and angiographic attempt at angiographic revascularization procedure was carried out. The femoral arteries were too calcified to pass the catheter through and the procedure was unsuccessful. Subsequently open femoral-popliteal bypass procedure was carried out, together with third and fourth rays amputation. Chlorine-based dressings were applied twice daily. On the ninth postoperative day, the wound was full of necrotic tissue and only a hint of granulation tissue observed. Antibiotic therapy was stopped at that time. Gradual improvement was seen with creeping substitution of the necrotic tissue with granulation tissue. At 14 weeks after beginning of COD dressing the patient came for a follow-up visit and the wound was closed. 

### 3.10. Case Report 10

**Figure 12:**
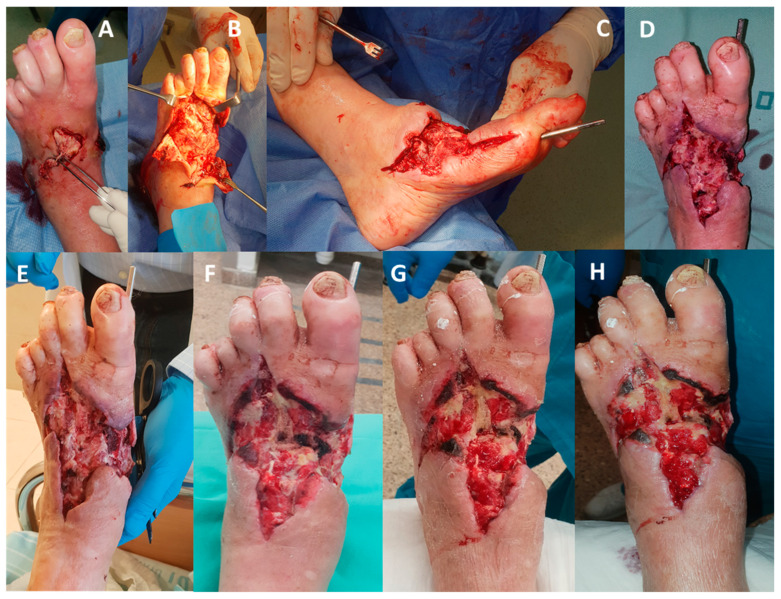
Impressive granulation despite lack of dorsal foot artery (Case Report 10). (**A**) Patient with sepsis and bacteremia due to necrotizing fasciitis, with diabetic midfoot deformity due to Charcot neuroarthropathy. (**B**) Patient underwent deep debridement to the bone. (**C**,**D**) The foot was stabilized with 5 mm Steinman Pin Beaming the medial column. (**D**) Following the operation, the wound was treated with chlorine-based solution (Milton solution) twice a day for four days. (**E**) One day after the operation the foot was viable with minor marginal skin necrosis. (**F**) Four days after the operation, the patients started to be treated with COD. (**G**) Three days after COD treatment, increase granulation is noted. (**H**) Ten days after COD treatment, extensive granulation is noted.

A 78-year-old diabetic female patient had diabetic midfoot deformity due to Charcot neuroarthropathy with ulceration and bone involvement. She developed sepsis and bacteremia due to necrotizing fasciitis. On admission she had necrotic areas of the skin (Figure 12A). There was bony deformity in the midfoot. She was operated on urgently. The deep structures including facia, tendons, and joint capsule were infected and necrotic (Figure 12B). The dorsalis pedis artery was necrotic as well. The infected tissues and involved bone were resected. The foot was stabilized with 5 mm Steinman Pin Beaming the medial column (Figure 12C,D). Post operatively the foot was dressed with chlorine-based solution (Milton solution) twice a day for four days. On post-operative day (PO-d) 1, the foot was viable with minor marginal skin necrosis (Figure 12E). On PO-d 4 the foot was stable with no granulation tissue (Figure 12F). COD dressing was initiated and changed every 3–4 days. The foot was splinted in a plaster slab. Intense granulation tissue seemed to take place from the first dressing change (COD d-3, PO-d-7) (Figure 12G) and increased thereafter (Figure 12H). In the meanwhile, the foot was stable with the Steinman pin. The patient was discharged home in a plaster of Paris cast with intent of weekly cast and dressing change until full granulation will be suitable for skin grafting. Upon discharge she developed extensive myocardial infarct and passed away. 

## 4. Discussion

In the current case series, we have described intense healing reaction in hard to heal wounds that were treated with copper oxide dressing (COD). Some of the wounds have had stagnation and nonhealing with other treatment modalities. Some of which had abundant fibrous and/or necrotic tissue, which was replaced by intense granulation that could be a favorable basis for skin grafting or re-epithelization. 

Today most antimicrobial wound dressings in the marketplace are silver-containing wound dressings. Antimicrobial wound dressings are widely used in wound treatment to reduce the risk of wound and wound-dressing contamination [39]. However, their usefulness in promoting wound healing is questionable, especially due to cellular toxicity [40,41]. Copper also has potent biocidal properties [18], but in contrast to silver, copper is an indispensable trace element extremely well metabolized by the human body [23], and could be a substitute for silver in wound dressings, if only to reduce biocontamination. More importantly, copper plays a key role in skin generation and angiogenesis [3,5,7,8,11,12,13,14,15,16,17], and has been shown to accelerate wound healing in animal models via induction of VEGF and angiogenesis [4,5,6]. Furthermore, in contrast to silver, which has been found to inhibit HIF-1α [42], copper enhances HIF-1α expression [4] and the binding of HIF-1α to the critical motifs in the promoter and putative enhancer regions of HIF-1 regulated genes [43]. HIF-1α has been recognized as a critical factor in wound healing [4,44]. We thus hypothesized that the inability of wounds to heal in individuals with compromised peripheral blood supply (e.g., with vascular diseases or diabetes), is partially due to low levels of copper in the wound site [1]. 

The above described 10 case reports clearly support our hypothesis that the copper oxide-containing wound dressing not only confer protection to the wound and the dressing from microbial contamination, and in some cases may help clear the wound infections (e.g., Case Reports 4 and 5), but in addition and more importantly, stimulate skin regeneration and wound healing. This is achieved via the constant release in situ of ppm of copper ions, which in the wound itself stimulate angiogenesis and formation of intense granulation tissue. This occurred even in some cases in which the blood supply to the wound is dramatically impaired, e.g., Case Report 6, of a patient suffering from PVD, and even more strikingly, as seen in Case Report 10, in which the dorsal artery and the necrotic dorsal structures (facia tendons and joint capsule) were removed in a 78-year-old diabetic patient, yet impressive granulation occurred shortly after the COD treatment. 

Fascinatingly, the wound healing kinetics observed were in some cases very similar, if not better, than the healing kinetics observed with VAC treatments (e.g., Case Reports 2 and 3). The improved wound healing was noted in patients with varied background diseases, such as patients with diabetes (IDDM and NIDDM), PVD, COPD, sickle cell disease, and renal failure, indicating that copper is a key player in the capacity of a wide array of hard to heal wounds to heal. 

The use of a control dressing and controls in general are very important in order to reach clear conclusions. The current article does not describe a controlled study but describes a series of case studies that were not a part of a trial, but were observations gathered as part of the standard of care. The current observations inspire us to continue studying the effect of the COD on wound healing and conduct controlled clinical studies to definitely establish the capacity of the copper oxide impregnated dressings to stimulate wound healing in hard to heal wounds.

## 5. Conclusions

Our results are in accordance with the results obtained in a murine diabetic model, in which the increased wound healing in the groups of mice treated with the copper oxide-containing dressings was not related to the copper potent biocidal properties, but to direct stimulation of wound repair by copper [1,4]. This may be of special importance especially in chronic wounds, such as diabetic wounds, venous and pressure ulcers, which fail to heal with other well-recognized wound care protocols. The copper dressings appear to hold significant promise in the clinician’s ongoing struggle to heal both acute and chronic wounds. Additional controlled studies should be conducted to further validate the efficacy and healing effect of topically applied copper-impregnated dressings.
